# Corneal biomechanical properties in healthy children measured by corneal visualization scheimpflug technology

**DOI:** 10.1186/s12886-017-0463-x

**Published:** 2017-05-17

**Authors:** Miao He, Hui Ding, Hong He, Chi Zhang, Liangping Liu, Xingwu Zhong

**Affiliations:** 1 Zhongshan Ophthalmic Center and State Key Laboratory of Ophthalmology, Sun Yat-sen University, 54S Xianlie Road, 510060 Guangzhou, China; 2 Hainan Eye Hospital, Zhongshan Ophthalmic Center, Sun Yat-sen University, Haikou, China

**Keywords:** Corvis ST, Corneal biomechanics, Corneal deformation, Symmetry, Chinese, Children

## Abstract

**Background:**

The aim of this study was to evaluate corneal biomechanical properties in a population of healthy children in China using corneal visualization Scheimpflug technology (CST).

**Methods:**

All children underwent complete bi-ocular examinations. CST provided intraocular pressure (IOP) and corneal biomechanical parameters, including time, velocity, length and deformation amplitude at first applanation (A1T, A1V, A1L, A1DA), at second applanation (A2T, A2V, A2L, A2DA), highest concavity time (HCT), maximum deformation amplitude (MDA), peak distance (PD), and radius of curvature (RoC). Pearson correlation analysis was used to assess the impacts of demographic factors, central corneal thickness (CCT), spherical equivalent (SE), and IOP on corneal biomechanics.

**Results:**

One hundred eight subjects (32 girls and 76 boys) with the mean age of 10.80 ± 4.13 years (range 4 to18 years) were included in the final analyses. The right and left eyes were highly symmetrical in SE (*p* = 0.082), IOP (*p* = 0.235), or CCT (*p* = 0.210). Mean A1T of the right eyes was 7.424 ± 0.340 ms; the left eyes 7.451 ± 0.365 ms. MDA was 0.993 ± 0.102 mm in the right eyes and 0.982 ± 0.100 mm in the left eyes. Mean HCT of the right eyes was 16.675 ± 0.502 ms; the left eyes 16.735 ± 0.555 ms. All CST parameters of both eye were remarkably symmetrical with the exception of A2L (*p* = 0.006), A1DA (*p* = 0.025). The majority of CST parameters of both eyes were significantly correlated with CCT and IOP (*p* < 0.05). However, age, SE, and sex exert little influence on the CST measurements.

**Conclusions:**

This study found interocular symmetry in corneal biomechanics in healthy children eyes. Several CST biomechanical parameters in children are modified by CCT and IOP.

## Background

Corneal biomechanical properties play an important role in maintaining the shape and transparency of the cornea [1]. Various factors influence the corneal biomechanics such as hydration, elasticity, viscosity, and the thickness of corneal stroma [2]. Among the five layers of cornea, the stroma was considered to be the primary load-carrying layer. A large number of studies demonstrated that the corneal biomechanics altered significantly in eyes with keratoconus, Fuchs corneal dystrophy, glaucoma, post refractive surgery [3, 4]. Increase knowledge of corneal biomechanics is essential for the diagnosis and treatment of above diseases and prediction of the risk of ectasia after refractive surgery.

Two commercial devices are available for characterizing the biomechanical properties of the cornea in vivo currently, i.e. the Ocular Response Analyzer (ORA, Reichert, Buffalo, New York, USA) and corneal visualization Scheimpflug technology (Corvis ST [CST], Oculus, Wetzlar, Germany). The ORA was introduced in 2004 based on bidirectional applanation tonometry, which provides intraocular pressure (IOP) and two corneal biomechanical parameters: corneal hysteresis (CH) and corneal resistance factor (CRF) [5–7]. However, the ORA was criticized for poor repeatability and the fact that it is easily affected by corneal morphological parameters [8, 9]. In contrast to the ORA, the newer CST was based on corneal deformation measurement using Scheimpflug imaging technology, which records the whole process of cornea deformation following an air puff and provides over ten biomechanical parameters.

Children are a special population whose physiology and pathology are greatly different with adults. Knowledge of corneal biomechanics in children can help to master the ocular geometry and refractive status of the child. In the previous study of corneal biomechanics achieved by ORA, researchers have found that lower CH to be significantly associated with longer AL, poor correlation has been reported in adult studies [10]. Besides, ORA parameters could both reflect anterior geometry such as iris concavity [6, 7] and posterior geometry, research have confirmed that in myopic children the average RNFL thickness and rim area correlated positively with CH whereas the average cup-to-disc area ratio correlated negatively with CH [11]. Considering the reliability and reproducibility of ORA parameter were poor compared with CST, thus it is necessary to investigate the relationship between CST parameter and corneal biomechanics in children. The symmetry of the paired organs has been used as a tool to evaluate certain disease. Understanding the normal range of differences between eyes will help inform analysis of what degree of asymmetry between eyes can be considered possibly pathologic. Although several studies have investigated the corneal biomechanics in adults [3, 4, 12–18], we did not found any reports on corneal biomechanical properties in children using CST and the symmetry of biomechanics parameters. To fill this gap, we performed a cross-sectional study to assess the corneal biomechanics in healthy Chinese children using CST and evaluate the inter-ocular symmetry and associations of CST parameters with other factors.

## Methods

### Subjects

This observational and cross-sectional study was completed at the Hainan Eye Hospital of the Zhongshan Ophthalmic Center in China from July to August, 2015. The study followed the tenets of the Declaration of Helsinki and the protocol was reviewed and approved by the Ethics Review Board of Hainan Eye Hospital of the Zhongshan Ophthalmic Center (HNEH2015-015). Informed consents were obtained from the subjects’ parents before examination.

Healthy children without ocular disease (except refractive errors) were recruited at the hospital setting. The children were composed of 1) children came to hospital for regular optometry screening; 2) hospital employees’ children; 3) children whose parents visiting the hospital. Subjects with any systemic and/or ocular diseases that influence corneal evaluation, such as diabetes, connective tissue disorders, dry eye, uveitis, corneal ulcers, glaucoma, cataract, or retinopathy were excluded from the study. The children who wore contact lenses were also excluded. The ocular examinations were performed under the assistance of their parents.

### Ocular and CST examinations

All children underwent complete bi-ocular examinations, including slit-lamp evaluation, auto refractometer (RM-8900, TOPCON, Japan), and CST imaging (Oculus Optikgeräte GmbH, Wetzlar, Germany). Demographic data were recorded such as date of birth, sex, history of disease.

**Fig. 1:**
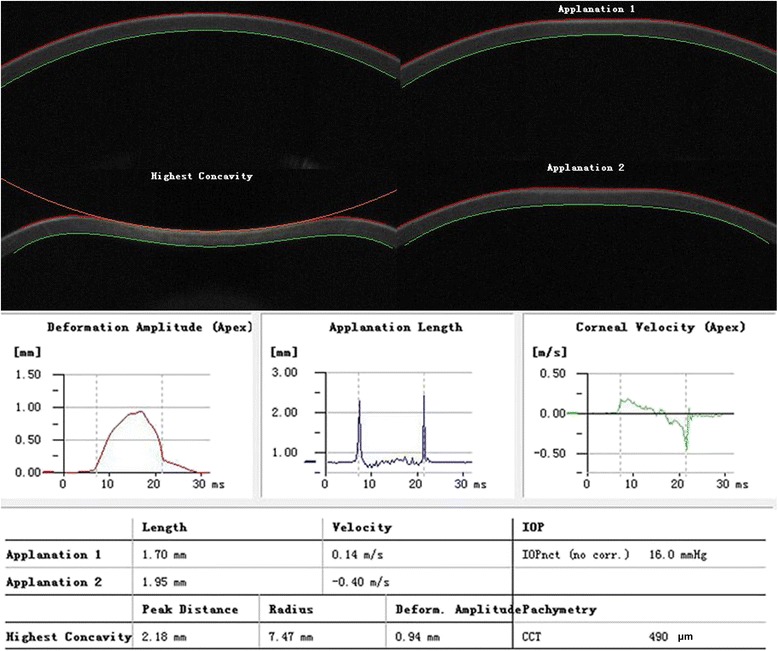
Diagram of corneal response parameters on the images from the corneal visualization Scheimpflug technology

All CST examinations were conducted by an experienced technician, who was blind to the study protocol. The right eye was firstly examined, then the left eye underwent CST scan. Children were asked to adjust their chin and forehead to an appropriate height, then focus on the red light emitting diode (LED). The exam is programmed for automatic release when alignment is achieved with the first Purkinje reflex of the cornea. The CST takes more than 4,300 frames of the central 8-mm horizontal portion per second with a high-speed Scheimpflug camera, which records and displays the entire response of cornea deformation to an air puff from the instrument. The air puff is 25 Kpa and lasts 20 ms. Finally, 140 digital frames are obtained. Each image has 576 possible measuring points. The cornea experiences four distinct statuses, i.e. first applanation (A1), the highest concavity (HC), second applanation (A2) and natural status (Fig. 1). The latest analyzing software (Corvis_ST_1.2r1126) was used in this study. Only the images with an “OK” in Quality Specification (QS) by built-in software were included in the final analysis.

### Statistical analysis

Statistical analysis was performed using SPSS 20.0 (SPSS, Inc., Chicago, IL). The Kolmogorov-Smirnov test was used to verify the distribution of normality. All results of continuous variables were presented as mean ± standard deviation (SD). The paired *t* test and intraclass correlation coefficient (ICC) were used to compare the measurements of the right and left eye. A Pearson correlation analysis was used to evaluate the relationships between age, gender, IOP, CCT, SE, and CST parameters. The level of significance was set at 5% with two sides.

## Results

Initially, a total of 140 children were invited for this study, but 20 refused to take part in this study. Among the 240 eyes (120 children), 40 eyes were excluded because inadequate quality of the CST images. Thus, 200 eyes of 108 children with 32 girls and 76 boys were included in the final analyses, with the mean age of 10.80 ± 4.13 years (range 4 to18 years). Table 1 summarizes the demographic and clinical data of the subjects. The right and left eyes were highly symmetrical in SE (*p* = 0.082), IOP (*p* = 0.235), or CCT (*p* = 0.210) (Table 1).

**Table 1:** Demographic and ocular characteristics of subjects

Variables	Mean ± SD	P*
Age (years)	10.80 ± 4.13	
SE (diopters)		0.082
Right eye	−3.13 ± 3.69	
Left eye	−2.82 ± 3.77	
IOP (mmHg)		0.235
Right eye	15.83 ± 2.84	
Left eye	16.03 ± 3.03	
CCT (μm)		0.210
Right eye	549.62 ± 30.96	
Left eye	550.83 ± 32.49	

*SE* spherical equivalent, *IOP* intraocular pressure, *CCT* central corneal thickness, *P* *P* values, *paired *t* test

The mean values of CST parameters are presented in Table 2. Mean A1T of the right eyes was 7.424 ± 0.340 ms; the left eyes 7.451 ± 0.365 ms. MDA was 0.993 ± 0.102 mm in the right eyes and 0.982 ± 0.100 mm in the left eyes. Mean HCT of the right eyes was 16.675 ± 0.502 ms; the left eyes16.735 ± 0.555 ms. All CST parameters of both eye were similar with the exception of A2L (*p* = 0.006), A1DA (*p* = 0.025). These results revealed that the right eyes and left eyes were highly symmetric in most CST parameters.

**Table 2:** Mean values and inter-eye symmetry of corneal biomechanical parameters in subjects

Parameters	Right eye	Left eye	Mean difference (95% CI)	P*	ICC
A1T, ms	7.424 ± 0.340	7.451 ± 0.365	−0.028 (-0.067 to 0.012)	0.167	0.906
A1L, mm	1.729 ± 0.066	1.726 ± 0.094	0.003 (-0.016 to 0.021)	0.784	0.415
A1V, m/s	0.139 ± 0.020	0.138 ± 0.026	0.001 (-0.004 to 0.005)	0.861	0.592
A1DA, mm	0.119 ± 0.009	0.116 ± 0.011	0.002 (0.001 to 0.004)	**0.025**	0.685
A2T, ms	21.586 ± 0.869	21.401 ± 1.216	0.184 (-0.097 to 0.466)	0.197	0.224
A2L,mm	1.688 ± 0.319	1.554 ± 0.383	0.133 (0.038 to 0.228)	**0.006**	0.199
A2V, m/s	−0.334 ± 0.102	−0.334 ± 0.078	0.0002 (-0.022 to 0.022)	0.985	0.451
A2DA, mm	0.367 ± 0.131	0.372 ± 0.141	−0.004 (-0.038 to 0.030)	0.800	0.396
HCT, ms	16.675 ± 0.502	16.735 ± 0.555	−0.060 (-0.197 to 0.077)	0.389	0.137
PD, mm	2.940 ± 1.083	2.686 ± 1.080	0.253 (-0.037 to 0.544)	0.086	0.027
RoC, mm	6.470 ± 1.295	6.438 ± 1.318	0.032 (-0.265 to 0.330)	0.831	0.449
MDA, mm	0.993 ± 0.102	0.982 ± 0.100	0.011 (-0.002 to 0.024)	0.088	0.872

Data were presented as mean ± standard deviation

*95% CI* 95% confidential interval, *paired *t* test, *ICC* intraclass correlation coefficient

*A1T* Time from start to the first flatterning of cornea, *A1L* Length of flattened cornea in the first applanation, *A1V* Inward velocity of the cornea in the first applanation, *A1DA* Cornea apex displacement in vertical direction at first applanation, *A2T* Time from start to second flattening of the cornea, *A2L* Length of flattened cornea in the second applanation, *A2V* Outward velocity of the cornea in the second applanation, *A2DA* Cornea apex displacement in vertical direction at second applanation, *HCT* Time from start to the highest concavity of cornea, *PD* Distance between the corneal peaks at maximal concavity, *RoC* Radius of curvature of the corneal at highest concavity, *MDA* Cornea apex displacement in vertical direction at highest concavity

Bold indicates statistical significance (*P* < 0.05)

Table 3 shows the results of correlation analyses. Age and gender were not significantly associated with most CST parameters in the right eyes or left eyes. CCT was significantly and positively correlated to A1T, A1DA of the both eyes. SE was positively correlated to HCT, A2DA of the right eye and MDA, PD, A2DA of the left eye. IOP was positively associated with A1T, A1L, A2V, RoC, and A1DA in both eyes, while negatively related to MDA, A1V, A2T of both eyes.

**Table 3:** Correlations between corneal biomechanical parameters and demographic or ocular parameters

	Age		CCT		Sex		SE		IOP	
	*r*	*P*	*r*	*P*	*r*	*P*	*r*	*P*	*r*	*P*
Right eye										
A1T, ms	0.071	0.464	0.272	**0.004**	0.145	0.131	−0.104	0.278	0.995	**<0.001**
A1L, mm	0.102	0.288	0.281	**0.003**	0.027	0.783	1.000	0.298	0.170	0.076
A1V, m/s	−0.035	0.719	−0.170	0.075	−0.043	0.655	0.047	0.626	−0.735	**<0.001**
A1DA, mm	−0.137	0.152	0.407	**<0.001**	−0.075	0.437	0.167	0.079	0.509	**<0.001**
A2T,ms	0.014	0.888	−0.253	**0.010**	−0.205	**0.038**	0.125	0.205	−0.355	**<0.001**
A2L, mm	0.018	0.857	0.089	0.371	−0.103	0.298	−0.065	0.511	0.148	0.134
A2V, m/s	0.027	0.788	0.114	0.252	−0.034	0.736	0.115	0.242	0.525	**<0.001**
A2DA, mm	−0.117	0.239	0.311	**0.001**	0.116	0.242	0.409	<0.001	−0.158	0.112
HCT, ms	0.014	0.882	0.063	0.513	0.076	0.429	0.234	**0.014**	−0.122	0.205
PD, mm	−0.282	**0.003**	−0.003	0.974	−0.131	0.173	−0.049	0.608	−0.149	0.120
RoC, mm	0.136	0.155	0.125	0.194	−0.021	0.824	0.070	0.468	0.319	**0.001**
MDA, mm	0.026	0.789	−0.139	0.147	−0.113	0.241	−0.071	0.456	−0.801	**<0.001**
Left eye										
A1T, ms	−0.038	0.696	0.333	**<0.001**	0.074	0.445	−0.041	0.668	0.995	**<0.001**
VA1L, mm	−0.078	0.425	0.104	0.286	−0.034	0.729	0.175	0.067	0.197	**0.041**
A1V, m/s	−0.077	0.426	0.033	0.735	−0.052	0.595	0.039	0.690	−0.444	**<0.001**
A1DA, mm	−0.083	0.395	0.426	**<0.001**	0.031	0.749	0.076	0.428	0.538	**<0.001**
A2T,ms	−0.005	0.957	−0.041	0.684	0.033	0.740	0.077	0.434	−0.383	**<0.001**
A2L, mm	0.153	0.123	0.249	**0.011**	−0.020	0.838	0.064	0.516	0.158	0.112
A2V, m/s	−0.026	0.801	0.193	0.055	0.154	0.126	0.100	0.315	0.458	**<0.001**
A2DA, mm	−0.091	0.370	0.026	0.798	0.093	0.355	0.233	**0.019**	−0.152	0.130
HCT, ms	−0.101	0.298	0.051	0.601	0.129	0.183	0.084	0.381	−0.119	0.218
PD, mm	0.074	0.448	−0.159	0.101	−0.122	0.208	−0.242	**0.011**	−0.193	**0.046**
RoC, mm	0.059	0.545	0.161	0.096	0.008	0.937	0.249	0.090	0.260	**0.007**
MDA, mm	0.078	0.421	−0.167	0.084	−0.042	0.667	−0.257	**0.007**	−0.798	**<0.001**

*IOP* Intraocular pressure, *CCT* Central corneal thickness, *SE* spherical equivalent

*A1T* Time from start to the first flatterning of cornea, *A1L* Length of flattened cornea in the first applanation, *A1V* Inward velocity of the cornea in the first applanation, *A1DA* Cornea apex displacement in vertical direction at first applanation, *A2T* Time from start to second flattening of the cornea, *A2L* Length of flattened cornea in the second applanation, *A2V* Outward velocity of the cornea in the second applanation, *A2DA* Cornea apex displacement in vertical direction at second applanation, *HCT* Time from start to the highest concavity of cornea, *PD* Distance between the corneal peaks at maximal concavity, *RoC* Radius of curvature of the corneal at highest concavity, *MDA* Cornea apex displacement in vertical direction at highest concavity

Bold indicates statistical significance (*P* < 0.05)

## Discussion

We evaluated the corneal biomechanical properties in a population of healthy children in China. As far as we know, this is the first report of quantitative assessment of the corneal biomechanics in children population using CST. We used the newly updated CST software, which provided two more parameters (A1DA and A2DA) than previous versions. This helped to measure corneal deformation more comprehensively. We also assessed the symmetry of corneal biomechanics between the both eyes and found obviously interocular symmetry in SE, CCT, IOP, and corneal biomechanics in healthy children eyes. We also found that several CST biomechanical parameters in children are modified by CCT and IOP, while age, SE, and sex exert little influence on the CST measurements in this population.

Our observations of interocular symmetric biometry were consistent with previous studies [19–21]. Using ORA, Zheng et al. [22] demonstrated an obvious symmetry of CH and CRF in bilateral rabbit corneas. We also identified two asymmetrical parameters in the sample, i.e. A2L and A1DA. The corneal stroma is composed of highly arranged collagen fibrils [23]. Wide-angle x-ray scattering study demonstrated that distribution of the aligned fibrils in the cornea generally was symmetrical in the both eyes, but the mass of preferentially aligned collagen in the corneal stroma appears to be distributed differently between left and right eyes [24]. Thus, we postulate that the asymmetry of A2L and A1DA may be affected by asymmetry arrangement of corneal collagen. Besides the poor repeatability and reproducibility of A2L and A1DA which were reported by previous studies [25–27] also could explain some of differences found between the right eye and the left eye. However, further studies are warrant to clarify the exactly mechanism.

One interesting finding is that age was not correlated with CST parameters in children. Previous studies have investigated children’s corneal biomechanics using ORA, but Huang et al. [10], Shah et al. [6, 7] and Buenoet al. [11] obtained controversial results. In Brazil adults, Valbon et al. [14] reported that age was positively correlated to HCT (*P* = 0.048). In Chinese adults, Wang and his colleagues also revealed a positively correlation between age and A2L (*p* = 0.03) [18]. An increase in cross-linking fibers in corneal stroma with age was observed using X-ray, suggesting a stiffer cornea in older people [28, 29] The inconsistency between the present study and studies on adults using CST indicating the influence of age on CST parameters depended on age levels.

We found the most influential factor of CST parameters was IOP. We showed a positive correlation between IOP and A1T, A1L, A2V, RoC, and A1DA, while it indicated a negative correlation between IOP and MDA, A1V, A2T of both eyes, and PD of left eyes. These results suggest that corneal deformability decreased with an increasing IOP. Previous ORA studies have shown that IOP has important influences on CH and CRF [5–7]. The recent studies using CST also found glaucoma patients with high IOP have less deformable corneas [4, 16, 18]. We demonstrated that CCT was significantly and positively correlated to A1T, A1DA of both eyes, which was generally consistent with the deformation properties in adults [3, 4, 13].

The relationship between SE and CST parameters was complex. In the right eye, SE has a positive correlation with HCT and A2DA. In the left eye, SE has a negative relationship with MDA and PD, whereas has a positive correlation with A2DA. In the Italian adults, Lanza et al. [12] reported that SE was not correlated to A1 and A2 parameters and MDA in the left eye. The discrepancy may be related to different ethnicity, version of CST software, or age distribution of subjects. It was reported that corneal deformation behavior were affected by composition of collagen fibrils, hydration, and other as-yet-unknown factors [30]. More studies with large sample size are warranted to clarify the correlation of SE with CST parameters.

Limitations should not be ignored in the present study. First, this is a cross-sectional study with relative small sample size, thus our results need to be further confirmed by longitudinal cohort studies with larger sample size. Second, diurnal variation may exist because CH and CRF of ORA showed significant diurnal variation [31]. Third, axial length (AL) was not measured. Longer AL was reported to be significantly associated with lower CH (*p* < 0.001) [10]. The association between CST parameters and AL should be assessed in future because AL changes with the aging of children and is closely related to the progress of myopia. Besides, asymmetry found between the right eye and left eyes could not be confirmed since it was possible that not the real asymmetry existed but the poor reliability and reproducibility of some CST parameters cause the difference. Fourth, the sample subjects were not population based. For a mean difference of A1DA of 0.002, it was estimated that 215 subjects were required based on power of 90% and alpha of 5%, with a precision (standard deviations of both eyes) of 0.009. Our 108 subjects was fewer than estimated, however, the difference in A1DA between eyes was statistically significant so that the small sample size instead strengthen our findings. Thus, even inclusion of larger subjects in future study, the same conclusions will be expected. Finally, whether correlations between CST biomechanical parameters and CST derived IOP/CCT may in part be driven by the same instrument deriving these measures remain unclear. Thus, the exact implications of CST parameters needed to be elucidated.

## Conclusions

The present study evaluated the corneal biomechanical properties in healthy children in China using CST. The corneal biomechanical properties were generally symmetric in bilateral eyes. CCT and IOP have shown to have important influence on CST biomechanical parameters, while age, SE, and sex exert little influence on the CST measurements in children. Further studies with large sample size are needed to assess the corneal biomechanics in children population.

## Abbreviations

A1: First applanation
A1DA: Deformation amplitude at first applanation
A1L: Length at first applanation
A1T: Time at first applanation
A1V: Velocity at first applanation
A2: Second applanation
A2DA: Deformation amplitude at second applanation
A2L: Length at second applanation
A2T: Time at second applanation
A2V: Velocity at second applanation
AL: Axial length
CCT: Central corneal thickness
CH: Corneal hysteresis
CRF: Corneal resistance factor
HC: The highest concavity
HCT: Highest concavity time
IOP: Intraocular pressure
LED: Light emitting diode
MDA: Maximum deformation amplitude
PD: Peak distance
RoC: Radius of curvature
SD: Standard deviation
SE: Spherical equivalent
